# Exploring predictive models to improve the accuracy of Housing Price Index forecasts in India’s real estate sector

**DOI:** 10.1371/journal.pone.0341026

**Published:** 2026-01-23

**Authors:** Nutan Singh, Kavitha Shanmugam

**Affiliations:** 1 Department of Management Studies, CEG Campus, Anna University, Guindy, Chennai, India; Loughborough University, UNITED KINGDOM OF GREAT BRITAIN AND NORTHERN IRELAND

## Abstract

This study aims to improve the predictive accuracy of the Housing Price Index (HPI) in India using the unrestricted MIDAS (U-MIDAS) model with novel predictors, including total digital payment value (TDP), consumer price index (CPI), and financial stress index (FSI). Quarterly HPI data from the Reserve Bank of India (RBI) and monthly data for other variables were used from October 2019 to September 2024. Correlation analysis indicates that HPI and digital payments are positively correlated. There is a weak relationship between HPI and FSI. HPI and CPI have a negative relationship. The U-MIDAS model outperformed other models. The study highlights the effectiveness of the U-MIDAS methodology for short-term forecasting. The study further demonstrates that the forecast accuracy obtained with the unrestricted mixed data sampling (U-MIDAS) regression exceeds that of the ARIMAX model.

## Introduction

The research employs the unrestricted Mixed Data Sampling (U-MIDAS) regression model to enhance Housing Price Index (HPI) forecasts in India. It combines time-series data at multiple frequencies [1] to analyse relationships between economic indicators and housing prices. By including digital payment values and the financial stress index as predictors, the study underscores India’s increasing financial digitalisation [2,3]. Digital payment values may reflect consumer spending trends, economic activity, and market liquidity, all of which influence housing prices. The rise in digital payments suggests greater financial inclusion and spending capacity, which could impact housing demand [4]. According to the World Bank’s Digital Progress Report (2023), digital financial services support economic participation. During the pandemic, digital payments surged by 64 per cent in volume and 23 per cent in value in 2021−22 as people adopted contactless payment methods. To meet the increasing demand for digital transactions, the payment infrastructure has been upgraded by installing more PoS terminals and QR codes [5]. Similarly, the financial stress index could provide a broader perspective on economic health and investor sentiment [6].

The Indian Housing Price Index (HPI) needs improvement to capture housing market dynamics better. Existing indices such as RESIDEX and RBI’s HPI rely on outdated methods [7] and cover only a few cities, limiting their national relevance. Data collection is inconsistent, as it uses population ratios instead of transaction volumes for weighting. These indices do not account for variations in housing quality nor distinguish between property types. Irregular updates hinder their ability to respond swiftly to market shifts, and an inadequate mix of economic indicators impairs analysis. The lack of standardised methods, transparency, and consideration of local markets undermines their reliability. An updated HPI would provide policymakers, investors, and stakeholders with a clearer picture of India’s housing trends.

Traditional models for predicting housing prices in India face numerous challenges, especially in a rapidly digitising economy and an evolving urban landscape. These models often fail to incorporate high-frequency data, such as digital payment values and financial stress indices, and usually overlook the growing influence of digital transactions and fintech on market dynamics. Furthermore, they struggle to adapt to frequent policy changes and urbanisation patterns, and they are unable to handle mixed-frequency data, which limits their ability to produce timely “nowcasts” of current market conditions. To address these issues, advanced techniques such as the Unrestricted Mixed Data Sampling (U-MIDAS) regression model are being explored for their ability to integrate diverse data streams and deliver more accurate, responsive forecasts tailored to India’s housing market.

This research aims to improve the accuracy of Housing Price Index (HPI) predictions in India by applying the unrestricted Mixed Data Sampling (U-MIDAS) regression model, marking its first application in this context. The study introduces novel predictors, such as total digital payment values and the financial stress index, alongside traditional indicators to better understand India’s housing price trends. By integrating high- and low-frequency data series, the proposed model enhances short-term forecasting accuracy.

Housing serves as a significant indicator of a nation’s progress in terms of human development and is regarded as a means of long-term wealth accumulation. The housing sector fuels macroeconomic employment and economic activity. [8]. Consequently, housing has consistently been a priority on governmental agendas worldwide, as it constitutes a vital sector of the national economy with numerous backward and forward linkages, resulting in a substantial multiplier effect on the overall economy [8]. India maintained its position as the fastest-growing major economy by registering year-on-year GDP growth of 7.6 per cent in Q2 FY24, driven primarily by the Manufacturing (13.9 per cent) and Construction sectors (13.3 per cent). The Indian real estate sector is propelled by a favourable domestic economic environment, characterised by economic resilience, infrastructure growth plans, alternative investment models, and domestic consumption power [8].

In India, the real estate sector ranks second among employment generators, behind agriculture [9]. The Indian real estate market is projected to reach US$5–7 trillion by 2047, with the potential to surpass US$10 trillion. The sector aims to reach US$1 trillion by 2030 and contribute 13% to GDP by 2025 [9]. Urbanisation trends indicate a population growth to 542.7 million by 2025 and 675.5 million by 2035. The construction industry is one of the largest sectors in terms of foreign direct investment (FDI) inflow. FDI in this sector, encompassing construction development and activities, amounted to Rs. 3,83,229.78 crore from April 2000 to September 2024. India’s ‘Housing for All’ initiative is expected to attract US$1.3 trillion in housing investment by 2025. The demand for residential space is projected to increase significantly, driven by key factors such as rapid urbanization, population growth, an increase in nuclear families, easy access to finance, repatriation of non-resident Indians (NRIs) and high-net-worth individuals (HNIs), and rising disposable incomes [9].[10] also suggest that India has experienced rise in demand for housing since 2001, owing to increase in levels of income, younger earning age group, rapid urbanisation and nuclearization of families.

Government initiatives like Digital India and Jan Dhan Yojana have incredibly advanced financial inclusion in India. Information and Communication Technology is increasingly providing access to the unbanked population, thereby facilitating their integration into the banking sector. Digital technologies enhance usage and impact citizens’ livelihoods. India’s reforms foster an environment for discussing digital finance, forecasting, and policy planning in real estate [2]. The payments ecosystem in India has experienced significant advancement, characterised by the emergence of diverse payment systems and platforms, as well as payment products and services tailored to various consumer categories, including individuals, businesses, corporations, government entities, and other economic agents [3].Economic recovery from the pandemic has been driven by digitalisation, which remains crucial to nations’ future resilience. Digital goods and services greatly influence national economic growth, providing a robust and vital framework, especially in India, where the digital economy spans multiple sectors [11].

Housing indices help to gauge the housing prices, reflecting a balance between demand and supply of houses in any country [7]. As a basic necessity, India’s growing population is in constant need of housing. Acquiring a home consumes a significant portion of household savings, and is a lifelong goal for many families. However, recent real estate price surges have made homeownership increasingly difficult for the average citizens [7]. [12] examined the impact of pre- and post-pandemic real estate housing price indices in Indian cities. This study examined the effects of COVID-19 on residential housing prices using interrupted time-series analysis across 10 Indian cities from 2010 to 2023. Findings showed varied city-level trends, with a slowdown after the pandemic, followed by a positive shift. The study highlighted diverse effects and resilience among cities. [13] found that actual price rises in India’s housing market occurred after the slowdown. The key lesson for India is to maintain financial stability and regulate credit. The Indian economy is shifting from low to high growth, with the government promoting the housing sector, which is connected to approximately 300 industries and linked to the credit market, banking institutions, and monetary policy.

Housing price indices are crucial for understanding housing price changes and economic growth trends, and should be publicly accessible and accurately calculated. In India, despite the existence of indices such as the National Housing Board’s RESIDEX and the Reserve Bank of India’s HPI, significant gaps remain in our understanding of real-time housing price movements owing to their reliance on outdated methodologies, limited city coverage, and data collection inconsistencies. RESIDEX relies on data from various banks and finance companies in 26 cities, whereas RBI’s HPI uses data from registration departments in ten cities to calculate a national HPI, which is a weighted average of city-level HPIs, ideally weighted by the number of transactions, but instead uses city population proportions due to data collection issues [7]. Divergent trends in RESIDEX and HPI complicate economic analysis. Data transformation requires precise indices, which are publicly available [7].

Calculating house prices remains difficult due to differences in quality. The hedonic price index is an effective adjustment method. [14] constructed a hedonic price index using the Time Dummy Method and the Characteristics Price Index method based on the rent and sale/resale prices of Mumbai residential properties from January 2004 to November 2007. The findings show that quality adjustment significantly affects the indices, with hedonic house price indices being considerably lower than traditional median-weighted average price indices. [15] investigated real estate boom-bust cycles in Japan from 1980 to 1990 and in the United States from 2007 to 2009. Economic growth in both countries led to lower interest rates and increased credit availability. This caused housing prices to rise. When interest rates increased, loan defaults grew, reducing bank lending and disrupting economic growth.

Fluctuations in the real estate market and housing bubbles trigger banking crises. The housing sector plays a vital role in India’s economy, being the second-largest employer after agriculture. It supports migrants and related industries [16].Housing prices differ across localities because of variations in price-to-income and rent ratios. Cities with a dense supply show higher price-to-rent ratios and are more sensitive to interest rate changes. Although future fundamental changes may lower prices, this does not necessarily imply current market mispricing.[17]. To predict housing values, [18] included variables such as location, size, number of rooms, type of area, availability, and sale prices and applied several well-known machine learning techniques. In their analysis, they used methods such as SVM, random forests, XGBoost, Lasso regression, and linear regression. In measuring house value, [19] included factors like location, neighbourhood, and garage space. The XGBoost regression method is utilized to calculate the house price. House prices fluctuate based on land values and infrastructure developments. [19] recommended a centralised system that considers neighbourhood and infrastructure factors. It would help in estimating a house’s value.

In India, precise housing price tracking is vital for economic stability, because of the importance and volatility of the housing market.[20] estimated methods for constructing a House Price Index for India and comparing similar houses over a period through quality-adjusted calculations. The hedonic method is a promising approach for estimating price trends of typical houses traded annually with improved accuracy through detailed market knowledge. However, the hedonic model’s limitations and India’s dynamic real estate market, with its significant variations in housing quality preferences, suggest that relying solely on the House Price Index for decision-making may be inadequate. [10] compared India’s housing sector with the US and Spanish markets, analysing property values, financial institutions, credit flows, and regulatory structures. Since 2001, housing demand has surged due to rising incomes, urbanisation, and the growth of nuclear families. Macroprudential measures effectively influence housing prices, as shown by data from Indian cities. Loan-to-value (LTV) ratios and risk requirements impact house prices, with LTV adjustments restraining speculation [21].[22] indicated a strong positive correlation between the House Price Index and various economic factors, such as GDP, Housing Credit, Exchange Rates, and Inflation, while interest rates showed a slight negative correlation. This suggests that house prices generally align with most economic variables except for interest rates. [23] studied housing prices in Russia, China, and India, considering GDP, interest rates, inflation, population density, and unemployment through linear regression. Population density is strongly connected to housing prices across all three countries. Russia’s house prices declined due to reduced inflation and a population decline. India’s housing market was significantly influenced by inflation.

Traditional forecasting methods that rely on infrequent data are insufficient for short-term prediction. In contrast, MIDAS modelling is suitable for short-term forecasting when different data series are available at various frequencies [1]. [24] analysed daily electricity consumption and other high-frequency data to forecast industrial value-added using the Mixed Data Sampling (MIDAS) model and observed improved short-term forecast accuracy. The MIDAS model outperformed ARDL in predicting China’s power sector. It offers higher accuracy and more timely nowcasting, especially when data release is delayed [25]. [26] proposed a MIDAS model that incorporates a series of different frequencies for short-term inflation forecasts. The MIDAS-Almon polynomial distributed lag method performed best based on the AIC and SIC criteria, demonstrating satisfactory predictive accuracy. [27] used the U-MIDAS model and wavelet analysis to reveal a significant causal relationship between oil prices and economic activity in five Asian economies from 1998Q1 to 2019Q4, which is less apparent using the standard VAR method. [28] highlighted the utility of MIDAS (Mixed Data Sampling) regression in addressing “ mixed-frequency forecasting challenges for economic growth nowcasting. They found that MIDAS models using quarterly GDP data and monthly data on inflation, industrial production, and the Philippine Stock Exchange Index outperformed traditional models. It exhibited higher accuracy in both in-sample and out-of-sample testing.[29] compared mixed-data sampling (MIDAS) and mixed-frequency VAR (MF-VAR) for nowcasting and forecasting quarterly euro area GDP growth using 20 monthly indicators. MF-VAR excels at long-term analysis, whereas MIDAS is better suited for short-term analysis. [30] used unrestricted mixed data sampling (U-MIDAS) regressions to enhance short-term real GDP growth forecasts in the euro area and Japan. The U-MIDAS model surpassed benchmarks and forecasters. [27] used U-MIDAS to analyse the relationship between oil prices and economic activity in five Asian economies. The key empirical results from the MIDAS approach showed a clear and significant causal link between oil prices and economic activity, whereas the standard VAR approach did not.

Despite the availability of models for predicting HPI, the accuracy and integration of high-frequency data, such as total digital payment (TDP) and financial stress index (FSI), remain underexplored. Our study addresses this gap by considering these novel variables to forecast the HPI. This research attempts to develop a suitable model for forecasting HPI in India by integrating innovative explanatory variables with traditional economic indicators within the MIDAS framework, offering policymakers and stakeholders in the housing sector insights.

This study specifically aims to: (i) evaluate the predictive power of the U-MIDAS model for HPI in India, (ii) investigate the relationship between HPI and variables such as TDP, CPI and FSI, and (iii) compare the forecast accuracy of U-MIDAS model with ARIMAX model. Accurate HPI forecasting is essential for the real estate industry. Unlike conventional models, U-MIDAS incorporates both high- and low-frequency data to enhance short-term housing price predictions [31].

### Conceptual framework of MIDAS regression model

Traditional time-series regression models use data sampled at uniform intervals, whereas regression models incorporating varying sampling frequencies remain underexplored. We examine methods to construct Mi(xed) Da(ta) S(ampling) regressions (MIDAS regressions), which address cases where high-frequency data provide valuable insights but the variable of interest is sampled less frequently.

MIDAS regressions are instrumental when data availability is limited. Macroeconomic data, such as price movements and monetary indicators, are accessible monthly, while GDP components are available quarterly. Therefore, the MIDAS approach combines monthly and quarterly data for forecasting. Nowcasting with high frequency indicators becomes relevant for policymakers because the publication lags for many low-frequency variables are quite substantial [1,32].

Time-series analysis requires datasets of the same frequency. In mixed-frequency data regression, high-frequency data must be aggregated to low-frequency data, leading to information loss. Mixed Data Sampling (MIDAS) regression [1] tackles this issue by considering different frequencies without modelling each predictor’s dynamics. Using high-frequency data to nowcast low-frequency variables improves forecasting accuracy.

The MIDAS prevents information loss from data aggregation and allows forecast updates with high-frequency data [33]. It is suitable for short-term predictions of low-frequency series such as inflation, economic growth, and the industrial production index.

For our initial approach, we examine the following foundational model, which incorporates variables from diverse frequencies:


$$y_{t}=\;X_{t,}^{`}\beta +f\left(Z_{t/m}\theta ,\lambda \right)+\;\varepsilon_{t}$$

In this equation, y_t_ represents the low-frequency dependent variable and X_t_ denotes a set of low-frequency regressors that correspond to y_t_. Z_t/m_ represents the group of high-frequency variables. To illustrate, if y_t_ has an annual frequency, m is 12 for a variable with a monthly frequency. The vectors β, λ, and θ are the parameters that need to be estimated. Because is customary, ε_t_ indicates the error term, which is assumed to be independent and identically distributed.

The individual coefficient method incorporates high-frequency variables into the model as independent variables. This approach can be illustrated in an expanded form as follows:


$$y_{t}=X_{t}^{`}\beta +\;\sum_{r=\text{0}}^{m-\text{1}}Z_{(t-r)/m}^{`}\theta_{r}+\;\varepsilon_{t}$$

In this context, Z`_(t-r)/m_ denotes the *I*^th^ delayed value of the high-frequency regressor Z., and it is evident that a distinct coefficient θ is calculated for each lagged term m of the Z regressor. This approach differs from the aggregation method in which the components of high-frequency variables are either summed or averaged to create a single regressor. In the latter case, one parameter λ was estimated instead of the individual parameters for each component.


$$y_{t}=X_{t}^{`}\beta +\left[\sum_{r=\text{0}}^{m-\text{1}}Z_{(t-r)/m}\right]\lambda +\;\varepsilon_{t}$$

The individual coefficient method assigns unique weights to each high-frequency term, which significantly increases the number of parameters, particularly in multivariate scenarios. In contrast, the aggregation approach reduces parameterization but imposes strict limitations on the coefficients. The MIDAS method offers various weighting schemes for the two extreme approaches. One of the most basic weighting schemes is the step function approach. In this method, the coefficients of the high-frequency variables are constrained using a step function.


$$y_{t}=X_{t}^{`}\beta +\;\sum_{r=\text{0}}^{k-\text{1}}Z_{(t-r)/m}^{`}\varphi_{r}+\;\varepsilon_{t}$$

The chosen number of lags for the high-frequency series, denoted by k, can be either greater or less than m. Although the number of parameters increases with the lag length of the high-frequency variable, this growth is slower than that in the individual coefficient method. Another approach involves employing the Almon delay function, which is a widely used technique in distributed lag modeling. This method expresses the coefficients of the delayed or lagged term (θ_r_) as polynomial varying weights. Consequently, the initial equation can be rewritten as


$$y_{t}=X_{t}^{`}\beta +\;\sum_{r=\text{0}}^{m-\text{1}}{Z`}_{(t-r)/m}\;\left[\sum_{j=\text{0}}^{p}{r^{j}\theta }_{j}\right]+\;\varepsilon_{t}$$

Where p is the order of the Almon polynomial.

An alternative method is the unrestricted MIDAS (U-MIDAS) model, which is suitable when variations in sampling frequencies are not substantial, such as with data collected on a monthly or quarterly basis [34]. This can be represented as follows:


$$y_{t}=X_{t}^{`}\beta +\;\sum_{r=\text{0}}^{m-\text{1}}\gamma_{(t-r)/m}\varphi_{(t-r)/m}+\;\varepsilon_{t}$$

In this approach, we calculated distinct slope coefficients for each high-frequency time lag.

When the dependent variable shows enough persistence and the frequency mismatch with the explanatory variables is minimal, there is compelling evidence that the U-MIDAS specification outperforms MIDAS. The primary challenges with MIDAS models arise from the non-linear restrictions, which complicate estimation. In contrast, U-MIDAS models can be easily estimated using OLS and do not require any common factor restrictions, resulting in a fast and straightforward estimation process [32].

This study presents a fresh approach by forecasting the Housing Price Index (HPI) using the U-MIDAS model. To the best of my knowledge, no prior research has used a MIDAS regression model to measure HPI. Most of the existing literature relies on traditional hedonic models and machine learning models. The hedonic price index principle is a widely recognised method for quality adjustment, whereas machine learning models depend on qualitative attributes. In contrast, MIDAS models are particularly suitable for mixed-frequency data, which is essentially quantitative.

### Predicting HPI using MIDAS regression approach

The study period spans from the third quarter of 2019 to the second quarter of 2024, with 2019Q3 to 2023Q2 designated as the training period and 2023Q3 to 2024Q2 as the testing period.We have projected the Housing Price Index (HPI) for the period from 2023Q3 to 2024Q2 and found that U-MIDAS outperformed all other MIDAS models under specific criteria.

This paper explores the use of the MIDAS model to forecast the Housing Price Index (HPI), employing predictors such as the Consumer Price Index (CPI), Total Digital Payment (TDP), and Financial Stress Index (FSI). It is distinctive in its application of mixed-frequency data: HPI data is available quarterly, whereas the other predictors are monthly. To my knowledge, this represents the first instance of employing these novel quantitative predictors for HPI forecasting.

Although real estate forecasting has been extensively researched in developed countries, there is a notable lack of empirical studies on Indian housing markets that utilise advanced forecasting techniques such as MIDAS modelling and machine learning. This paper seeks to address this gap by investigating the advanced MIDAS model for HPI prediction. The study aims to bridge this research gap and to contribute to the academic literature in the Indian context. It provides valuable insights for Indian policymakers, developers, and financial institutions.

### Variables and data

This study employs the total digital payment value (TDP), consumer price index (CPI), and financial stress index (FSI) to predict the housing price index (HPI) in the Indian economy. Because of differing data frequencies between the dependent and independent variables, MIDAS modelling was utilized.

The secondary data for the Housing Price Index (HPI) has been obtained from the Reserve Bank of India (RBI) database, which provides the All-India Housing Price Index based on data from ten cities across India [35].The Reserve Bank’s House Price Index (HPI) is derived from data on house transactions recorded at the time of registration, with information sourced from the registration departments of the respective state governments. This index is constructed from registration price data and calculated as a stratified weighted average, stratified by administrative zones within each city. To create the all-India index, the price indices for each city are averaged according to the population proportion (from the 2011 census) of ten cities—Mumbai, Delhi, Chennai, Kolkata, Bengaluru, Lucknow, Ahmedabad, Jaipur, Kanpur, and Kochi—relative to the total. The ten-city average HPI, compiled using the Laspeyres method, represents a weighted average of the city-level HPIs. These indices rely on official data from the registration authorities of various state governments and are compiled at both the city and national levels [36].

TDP includes credit/debit transfers, direct debits, card payments, and prepayment tools. CPI assesses consumer price changes using a representative basket of goods and services. It serves as a key inflation indicator for stakeholders. We obtained TDP and CPI data from the RBI database.

The FSI presents combined economic and financial indicators and illustrates their overall impact on the market and economy. The Financial Stress Index serves as a comprehensive indicator of the stability of the financial system [6], thereby affecting both investor confidence and the availability of credit within the housing sector.

The Financial Stress Index assesses the stability of the financial system. It influences both investor confidence and credit access.[37,38]. To calculate the FSI, Principal Component Analysis (PCA) [39] was applied to variables including the NSE50 index (Nifty), Bank Nifty Index (B_Nifty), Nifty financial services index (NFS), Nifty commodity index (Nifty_Comm), Indian imports (IMP), and Mumbai Interbank Overdraft Rate (MIBOR). Secondary data relevant to the variables used to develop the FSI were sourced from the National Stock Exchange of India, with data collected monthly. Interest and mortgage rates were excluded from the analysis due to their limited and inconsistent impact [21,22] on housing prices in India, with broader indicators like FSI offering a more comprehensive view of financial conditions, especially given the country’s low mortgage penetration.

For MIDAS modelling, stationarity is required across all variables. So, we conducted a unit root test (Augmented Dickey-Fuller (ADF)) test, using EViews software. Our findings indicate that the House Price Index (HPI) is stationary at the second difference. At the same time, the Total Digital Payment (TDP) and Consumer Price Index (CPI) are stationary at the first difference. In contrast, the Financial Stress Index (FSI) is stationary at a level. Consequently, we transformed the data for the selected variables accordingly.

## Results

In Table 1 (Correlation Matrix), DDHPI, DCPI, DTDP and FSI represent the stationary data series of HPI, CPI, TDP and FSI, respectively. HPI data is stationary at the second difference (DDHPI); CPI data is stationary at the first difference (DCPI); TDP is also stationary at the first difference (DTDP); and FSI data is stationary at the level. It is essential to have stationary time series data before conducting a correlation analysis.

**Table 1:** Correlation matrix.

	DDHPI	DCPI	DTDP	FSI
**DDHPI**	1.000000	-0.224810	0.491460	0.023078
**DCPI**	-0.224810	1.000000	-0.674393	-0.348193
**DTDP**	0.491460	-0.674393	1.000000	0.065460
**FSI**	0.023078	-0.348193	0.065460	1.000000

Note: Auther’s calculation.

The output of the quarterly correlation matrix (Table 1) shows the key relationships between DDHPI and the predictors. Digital payment data (DTDP) shows a positive relationship with DDHPI, indicating that digital financial activity influences housing price behaviour. The consumer price index (CPI) exhibits a weak negative correlation, suggesting that the housing price index may diverge from CPI trends. The financial stress index (FSI) shows a very weak positive correlation, and its nonlinear or lagged effects warrant further investigation.

In India, increases in consumer prices, as indicated by the Consumer Price Index (CPI), can have a notable impact on housing prices, as shown by the Housing Price Index (HPI), through several mechanisms. Rising CPI drives up living costs, reducing consumers’ disposable income and housing demand, especially among middle- and lower-income households. This causes a decline in housing prices. The RBI may adopt a more aggressive monetary policy by increasing interest rates to control inflation. Higher interest rates make home loans more expensive and lower housing affordability. During periods of high inflation, consumers tend to prioritise essential goods over residential property investments. This negatively impacts housing-sector demand and slows HPI growth.

This research evaluated and empirically compared various models (equations) based on their ability to effectively track the actual HPI. There are four models in the forecasting evaluation framework. EQ01 is a straightforward OLS model that considers quarterly data. EQ02, the MIDAS-PDL model, employs a polynomial lag structure for monthly predictors. EQ03, the MIDAS-Step model, uses step-wise weights to account for regime changes. EQ05, the U-MIDAS model, allows flexible lag coefficients to capture complex monthly effects. All the above equations are well explained in the ‘Conceptual Framework of the MIDAS Regression Model’ section of this research paper. These models explore housing price dynamics across different frequency data for the analysed period.

**Fig 1:**
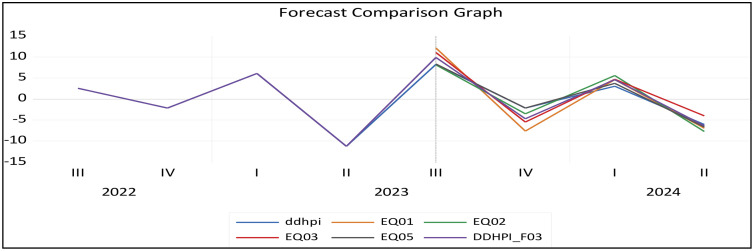
Forecasting averaging.

Fig 1 represents forecast comparison of EQ01, EQ02, EQ03 and EQ05 models. To combine these forecasts, a simple averaging method is used. It covers a one-year timeframe (forecast period), specifically from the third quarter of 2023 to the second quarter of 2024.

Table 2 presents a comparison of the forecast evaluations for all the four models, assessing their forecasting capabilities using different evaluation statistics.

**Table 2:** Forecast evaluation.

Forecast Evaluation						
Sample: 2019Q3 2024Q2						
Evaluation sample: 2019Q3 2024Q2						
Training sample: 2023Q3 2024Q2						
Combination tests						
Null hypothesis: Forecast i includes all information contained in others						
Equation	F-stat	F-prob				
EQ01	8.692988	0.002				
EQ02	1.115173	0.3786				
EQ03	10.39506	0.0009				
EQ05	0.112119	0.9515				
Evaluation statistics						
Forecast	RMSE	MAE	MAPE	SMAPE	Theil U1	Theil U2
EQ01	2.745297	2.100595	44.98622	39.43691	0.216427	0.251826
EQ02	1.170065	0.928272	24.87553	26.46644	0.085541	0.172913
EQ03	3.507718	2.714201	52.09866	49.92044	0.293097	0.334724
EQ05	0.257487	0.181612	5.23565	5.285836	0.018467	0.03918
Simple mean	1.614703	1.207892	23.33164	22.08694	0.125225	0.123328
Simple median	1.467903	1.023622	18.74462	18.13367	0.112736	0.1047
Least-squares	NA	NA	NA	NA	NA	NA
Mean square error	0.487199	0.403117	7.99974	7.786761	0.035639	0.051798
MSE ranks	1.580177	1.199926	23.22365	21.92589	0.122542	0.126339
Smooth AIC weights	2.499348	1.882723	36.79413	34.09122	0.201928	0.193499
SIC weights	2.459203	1.856429	36.19078	33.6283	0.198435	0.192062

Note: Auther’s calculation.

The forecast evaluation results, shown in Table 2, demonstrate the superior performance of the U-MIDAS model across various evaluation criteria. Its excellence is supported by the lowest RMSE, MAE, MAPE, and SMAPE values. It provides more accurate predictions and indicates better overall model performance.

**Fig 2:**
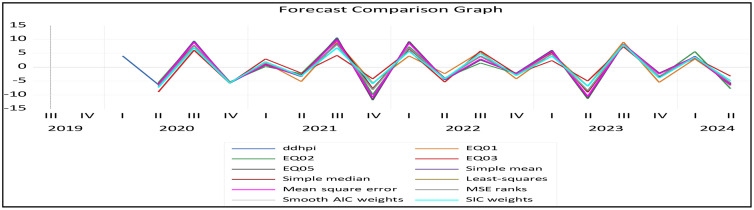
Forecast Evaluation of all models.

Fig 2 displays estimates of forecasts for all models over the studied period. It illustrates the forecast’s accuracy and reliability using specific criteria (RMSE, MAE, MAPE, and SMAPE).

Although various models, including basic OLS and other MIDAS variants, were assessed, the U-MIDAS model proved most effective at predicting HPI. Its outstanding performance across evaluation metrics (RMSE, MAE, MAPE, SMAPE) clearly indicates its effectiveness. It produces lower error rates. U-MIDAS should perform better than the original MIDAS as long as the aggregation frequency is minor and U-MIDAS is not too heavily parameterized [32]. The U-MIDAS (Unrestricted Mixed Data Sampling) model successfully forecasts using data of different frequencies. It is mainly designed for forecasting quarterly outcomes like GDP from monthly indicators [29,30]. U-MIDAS has a solid theoretical foundation for understanding forecasting challenges. We further demonstrate that the forecast accuracy obtained through the mixed data sampling (MIDAS) regression surpasses that achieved with ARIMAX model.

### Comparison of U-MIDAS model with ARIMAX model

These models capture linear relationships and require careful specification of lags and stationarity. It is frequently used in economic forecasting. They improve forecasts by including external regressors beyond the univariate methods. U-MIDAS handles mixed-frequency data series, while ARIMAX incorporates variables of the same frequency.

The ARIMAX model differs from the standard ARIMA model in its ability to incorporate exogenous variables. ARIMAX and most time-series models require stationary data to generate accurate results. ARIMAX integrates external variables into time-series forecasts when external factors influence the primary series. The model enhances forecast accuracy and offers a clearer insight into the data’s underlying dynamics [40].

The ARIMAX model demonstrates a statistically significant lagged effect of digital payment (DTDP(−3)) and a notable short-term impact of the financial stress index (FSI(−1)) on the house price index (DHPIM). As CPI does not show any link with HPI in correlation analysis (Table 1), so it is not included in ARIMAX model. Digital payment data (DTDP) shows moderate positive correlation with DDHPI, suggesting digital financial activity influences household price behavior, making it a key exogenous variable. Including MA [1] enhances the model’s fit and residual behaviour. The overall model is significant as the F-statistic falls within the acceptable range (p < 0.001). Therefore, this model is appropriate for short-term forecasting and offers valuable insights for policy development.

**Fig 3:**
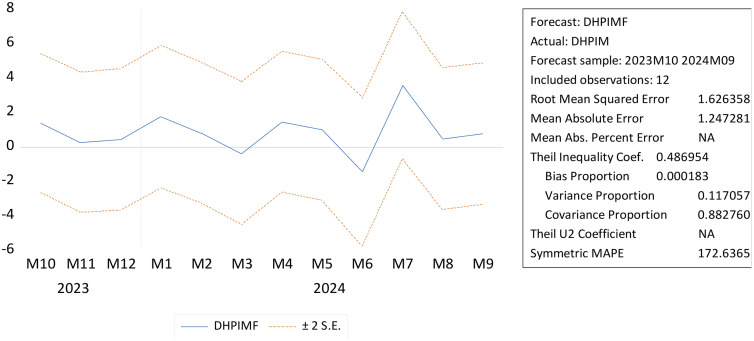
Forecasting HPI using ARIMAX model.

The analysis results show notable performance differences between U-MIDAS-based EQ05 forecast metrics (Table 2) and ARIMAX-based DHPIMF forecast metrics (Fig 3). EQ05 demonstrates superior accuracy with lower error measures, such as RMSE (0.257 vs. 1.626), MAE (0.182 vs. 1.247), and SMAPE (5.29% vs. 172.64%). Theil’s U1 statistic supports this, with EQ05’s value (0.0185) being closer to zero than DHPIMF’s (0.487). U-MIDAS effectively utilises mixed-frequency data without losing information through aggregation. The ARIMAX-based DHPIMF model shows the least systematic bias (0.0002) and the strongest directional traceability (covariance proportion 0.883). Consequently, U-MIDAS forecast evaluation metrics produce better results than ARIMAX forecast evaluation metrics.

## Discussion

Fluctuations in real estate values influence perceptions of household wealth, thereby affecting consumption and borrowing. Price-monitoring metrics are crucial for assessing property values and economic growth, and they must be precise and publicly accessible. Our investigation uses the unrestricted MIDAS (U-MIDAS) framework to predict India’s Housing Price Index (HPI). MIDAS is useful for short-term forecasts using multiple information intervals.

MIDAS models are instrumental in various academic disciplines where discrepancies in data frequencies occur as [41] considered the Ghysels Beta-MIDAS approach to evaluate tourism elasticity and found that the specific beta-coefficients of MIDAS indicated that the influence of tourism growth diminishes rapidly. Researchers used the MIDAS model to assess tourism’s impact on Tonga’s economy.

In a similar manner, [25] utilized the mixed-data sampling (MIDAS) regression model, incorporating variables of varying frequencies, to predict the annual carbon emissions of China’s power sector, and compared its performance to that of a standard model. The MIDAS model performed better for attaining predictive accuracy as compared the autoregressive distributed lag (ARDL) models.

(30) incorporate unrestricted mixed-data sampling (U-MIDAS) regressions for the evaluation of the effectiveness of various indicators in forecasting short-term real GDP growth in the euro area and Japan. Their results indicate that integrating predictions from multiple indicators enhances forecast accuracy and serves as an effective strategy to mitigate the volatility associated with monthly indicators.

Therefore, it is evident that the U-MIDAS approach is suitable when sampling frequency disparities are minimal, such as in monthly or quarterly data collection.

The study’s findings indicate that HPI has a positive association with digital transactions and a very weak correlation with FSI. In contrast, it demonstrates an opposite relationship with CPI.

The U-MIDAS model clearly surpassed the other MIDAS models as well as ARIMAX model for nearly all evaluation metrics. Lower RMSE, MAE, MAPE, and SMAPE values indicate improved predictions. The U-MIDAS model offers more accurate forecasts and is preferred for precise magnitude prediction.

### Policy and practical implications

Banks can enhance services by using AI and machine learning to analyse data and offer customised loans and insurance based on an individual’s credit scores [2].

The goal of offering a fundamental suite of financial services can be realized by banks through the design and development of tailored financial products. This can be achieved by effectively utilizing FinTech advancements to ensure efficient service delivery [42].

Micro-prudential and macro-prudential measures are vital for tackling financial stability issues caused by asset price bubbles, especially within a strong macroeconomic framework [43].

In addition to the recommendations as mentioned earlier, we wish to draw attention to several other suggestions that could enhance the housing price index and its significant role in overall economic growth such as, (i) Introduce national campaigns to attract real estate stakeholders. (ii) Organise workshops on digital payment systems. (iii) Provide guidelines for digital payments in real estate. (iv) Introduce tax incentives for digital transactions. (v) Enforce data privacy regulations. (vi) Form partnerships with fintech firms for real estate payment solutions. (vii) Mandate documentation of digital payments for high-value transactions. (viii) Link payment systems with tax and anti-money laundering frameworks. (ix) Digitise property registration with payment data. (x) Develop AI analytics platforms to analyse diversified macroeconomic data.

### Limitations of the study

(i) Housing registration data quality varies among cities. It impacts HPI accuracy. Digital payment figures and financial indicators as predictors may not fully capture local real estate markets.
(ii) The U-MIDAS model’s performance in other economic environments or real estate markets may differ significantly.
(iii) This study’s focus on the Indian real estate market limits the applicability of its findings to other markets with different economic, regulatory, and cultural factors.
(iv) The research spans October 2019 to September 2024, a period that includes significant global economic disruptions, notably the COVID-19 pandemic. While the study period allows for examining the impact of these disruptions on the real estate market, it also introduces volatility that may not be present in other time frames.
(v) Considering that the study spans five years with quarterly data, this duration might be inadequate to determine any causal relationships among the variables.

## Conclusion

This study demonstrates the effectiveness of the U-MIDAS model in forecasting India’s Housing Price Index (HPI). It surpasses other models, including the ARIMAX model. U-MIDAS is designed to handle mixed-frequency time series, whereas ARIMAX assumes all variables are of the same frequency. By incorporating predictors such as digital payment value and the financial stress index, the research offers insights into the Indian housing market. The findings show that lower RMSE, MAE, MAPE, and SMAPE values for the U-MIDAS model indicate superior forecast accuracy compared to the ARIMAX model. The U-MIDAS model provides more precise forecasts. However, policymakers need to address issues related to the consistency of real estate data, data privacy, digital payment platforms, digital literacy, and digital infrastructure challenges. Future research should focus on refining the U-MIDAS methodology and exploring additional high-frequency regressors to enhance HPI forecasting accuracy in India’s housing sector, thereby aiding decision-making in the real estate industry.

## Supporting information

S1 File Data. (XLSX)

S2 File Unit root test results. (DOCX)

S3 File ARIMAX model. (DOCX)
