# Supplementary material for: Exploring predictive models to improve the accuracy of Housing Price Index forecasts in India’s real estate sector
Source: PLoS One. 2026 Jan 23;21(1):e0341026. doi: 10.1371/journal.pone.0341026 (PMC12829794; doi:10.1371/journal.pone.0341026)
Supplement: S2 File — (DOCX) [file pone.0341026.s002.docx]

**Unit Root Test of all the variables**

1. **HPI**
2. **Stationary test at level**

| Null Hypothesis: HPI has a unit root | | | | |
| --- | --- | --- | --- | --- |
| Exogenous: Constant | | | |  |
| Lag Length: 3 (Automatic - based on SIC, maxlag=4) | | | | |
|  |  |  |  |  |
|  |  |  |  |  |
|  |  |  | t-Statistic | Prob.* |
|  |  |  |  |  |
|  |  |  |  |  |
| Augmented Dickey-Fuller test statistic | | | 2.720787 | 0.9999 |
| Test critical values: | 1% level |  | -3.920350 |  |
|  | 5% level |  | -3.065585 |  |
|  | 10% level |  | -2.673460 |  |
|  |  |  |  |  |
|  |  |  |  |  |
| *MacKinnon (1996) one-sided p-values. | | | | |
| Warning: Probabilities and critical values calculated for 20 observations | | | | |
| and may not be accurate for a sample size of 16 | | | | |
|  |  |  |  |  |
|  |  |  |  |  |
| Augmented Dickey-Fuller Test Equation | | | | |
| Dependent Variable: D(HPI) | | | | |
| Method: Least Squares | | | |  |
| Date: 05/30/25 Time: 21:29 | | | | |
| Sample (adjusted): 2020Q3 2024Q2 | | | | |
| Included observations: 16 after adjustments | | | | |
|  |  |  |  |  |
|  |  |  |  |  |
| Variable | Coefficient | Std. Error | t-Statistic | Prob. |
|  |  |  |  |  |
|  |  |  |  |  |
| HPI(-1) | 0.166198 | 0.061085 | 2.720787 | 0.0199 |
| D(HPI(-1)) | -1.258047 | 0.259443 | -4.849038 | 0.0005 |
| D(HPI(-2)) | -0.932565 | 0.338147 | -2.757873 | 0.0186 |
| D(HPI(-3)) | -0.602710 | 0.238786 | -2.524056 | 0.0283 |
| C | -40.17047 | 17.05825 | -2.354899 | 0.0382 |
|  |  |  |  |  |
|  |  |  |  |  |
| R-squared | 0.763865 | Mean dependent var | | 2.721668 |
| Adjusted R-squared | 0.677998 | S.D. dependent var | | 3.774948 |
| S.E. of regression | 2.142104 | Akaike info criterion | | 4.611760 |
| Sum squared resid | 50.47469 | Schwarz criterion | | 4.853194 |
| Log likelihood | -31.89408 | Hannan-Quinn criter. | | 4.624124 |
| F-statistic | 8.895876 | Durbin-Watson stat | | 1.702547 |
| Prob(F-statistic) | 0.001856 |  |  |  |
|  |  |  |  |  |
|  |  |  |  |  |

1. **Stationary test at first Difference**

| Null Hypothesis: D(HPI) has a unit root | | | | |
| --- | --- | --- | --- | --- |
| Exogenous: Constant | | | |  |
| Lag Length: 3 (Automatic - based on SIC, maxlag=4) | | | | |
|  |  |  |  |  |
|  |  |  |  |  |
|  |  |  | t-Statistic | Prob.* |
|  |  |  |  |  |
|  |  |  |  |  |
| Augmented Dickey-Fuller test statistic | | | -1.260519 | 0.6185 |
| Test critical values: | 1% level |  | -3.959148 |  |
|  | 5% level |  | -3.081002 |  |
|  | 10% level |  | -2.681330 |  |
|  |  |  |  |  |
|  |  |  |  |  |
| *MacKinnon (1996) one-sided p-values. | | | | |
| Warning: Probabilities and critical values calculated for 20 observations | | | | |
| and may not be accurate for a sample size of 15 | | | | |
|  |  |  |  |  |
|  |  |  |  |  |
| Augmented Dickey-Fuller Test Equation | | | | |
| Dependent Variable: D(HPI,2) | | | | |
| Method: Least Squares | | | |  |
| Date: 05/30/25 Time: 21:30 | | | | |
| Sample (adjusted): 2020Q4 2024Q2 | | | | |
| Included observations: 15 after adjustments | | | | |
|  |  |  |  |  |
|  |  |  |  |  |
| Variable | Coefficient | Std. Error | t-Statistic | Prob. |
|  |  |  |  |  |
|  |  |  |  |  |
| D(HPI(-1)) | -1.111900 | 0.882097 | -1.260519 | 0.2361 |
| D(HPI(-1),2) | -0.579551 | 0.725052 | -0.799323 | 0.4427 |
| D(HPI(-2),2) | -0.761354 | 0.512251 | -1.486292 | 0.1680 |
| D(HPI(-3),2) | -0.565574 | 0.244800 | -2.310357 | 0.0435 |
| C | 3.325262 | 2.196820 | 1.513671 | 0.1611 |
|  |  |  |  |  |
|  |  |  |  |  |
| R-squared | 0.922846 | Mean dependent var | | -0.440577 |
| Adjusted R-squared | 0.891985 | S.D. dependent var | | 7.058159 |
| S.E. of regression | 2.319711 | Akaike info criterion | | 4.781964 |
| Sum squared resid | 53.81057 | Schwarz criterion | | 5.017980 |
| Log likelihood | -30.86473 | Hannan-Quinn criter. | | 4.779449 |
| F-statistic | 29.90286 | Durbin-Watson stat | | 1.824796 |
| Prob(F-statistic) | 0.000015 |  |  |  |
|  |  |  |  |  |
|  |  |  |  |  |

1. **Stationary test at second Difference**

| Null Hypothesis: D(HPI,2) has a unit root | | | | |
| --- | --- | --- | --- | --- |
| Exogenous: Constant | | | |  |
| Lag Length: 4 (Automatic - based on SIC, maxlag=4) | | | | |
|  |  |  |  |  |
|  |  |  |  |  |
|  |  |  | t-Statistic | Prob.* |
|  |  |  |  |  |
|  |  |  |  |  |
| Augmented Dickey-Fuller test statistic | | | -3.990729 | 0.0112 |
| Test critical values: | 1% level |  | -4.057910 |  |
|  | 5% level |  | -3.119910 |  |
|  | 10% level |  | -2.701103 |  |
|  |  |  |  |  |
|  |  |  |  |  |
| *MacKinnon (1996) one-sided p-values. | | | | |
| Warning: Probabilities and critical values calculated for 20 observations | | | | |
| and may not be accurate for a sample size of 13 | | | | |
|  |  |  |  |  |
|  |  |  |  |  |
| Augmented Dickey-Fuller Test Equation | | | | |
| Dependent Variable: D(HPI,3) | | | | |
| Method: Least Squares | | | |  |
| Date: 05/30/25 Time: 21:30 | | | | |
| Sample (adjusted): 2021Q2 2024Q2 | | | | |
| Included observations: 13 after adjustments | | | | |
|  |  |  |  |  |
|  |  |  |  |  |
| Variable | Coefficient | Std. Error | t-Statistic | Prob. |
|  |  |  |  |  |
|  |  |  |  |  |
| D(HPI(-1),2) | -7.485507 | 1.875724 | -3.990729 | 0.0053 |
| D(HPI(-1),3) | 5.056113 | 1.697983 | 2.977718 | 0.0206 |
| D(HPI(-2),3) | 3.335025 | 1.269604 | 2.626822 | 0.0341 |
| D(HPI(-3),3) | 1.754478 | 0.744312 | 2.357182 | 0.0506 |
| D(HPI(-4),3) | 0.740854 | 0.278745 | 2.657824 | 0.0326 |
| C | 0.905377 | 0.580497 | 1.559659 | 0.1628 |
|  |  |  |  |  |
|  |  |  |  |  |
| R-squared | 0.988424 | Mean dependent var | | -0.534573 |
| Adjusted R-squared | 0.980156 | S.D. dependent var | | 14.13153 |
| S.E. of regression | 1.990703 | Akaike info criterion | | 4.518890 |
| Sum squared resid | 27.74028 | Schwarz criterion | | 4.779636 |
| Log likelihood | -23.37279 | Hannan-Quinn criter. | | 4.465295 |
| F-statistic | 119.5418 | Durbin-Watson stat | | 1.815222 |
| Prob(F-statistic) | 0.000001 |  |  |  |
|  |  |  |  |  |
|  |  |  |  |  |

1. **TDP (Total Digital Payment)**
2. **Stationary test at level**

| Null Hypothesis: TDP_VALUE_ has a unit root | | | | |
| --- | --- | --- | --- | --- |
| Exogenous: Constant | | | |  |
| Lag Length: 3 (Automatic - based on SIC, maxlag=10) | | | | |
|  |  |  |  |  |
|  |  |  |  |  |
|  |  |  | t-Statistic | Prob.* |
|  |  |  |  |  |
|  |  |  |  |  |
| Augmented Dickey-Fuller test statistic | | | 1.215214 | 0.9979 |
| Test critical values: | 1% level |  | -3.552666 |  |
|  | 5% level |  | -2.914517 |  |
|  | 10% level |  | -2.595033 |  |
|  |  |  |  |  |
|  |  |  |  |  |
| *MacKinnon (1996) one-sided p-values. | | | | |
|  |  |  |  |  |
|  |  |  |  |  |
| Augmented Dickey-Fuller Test Equation | | | | |
| Dependent Variable: D(TDP_VALUE_) | | | | |
| Method: Least Squares | | | |  |
| Date: 05/30/25 Time: 21:23 | | | | |
| Sample (adjusted): 2020M02 2024M09 | | | | |
| Included observations: 56 after adjustments | | | | |
|  |  |  |  |  |
|  |  |  |  |  |
| Variable | Coefficient | Std. Error | t-Statistic | Prob. |
|  |  |  |  |  |
|  |  |  |  |  |
| TDP_VALUE_(-1) | 0.093644 | 0.077060 | 1.215214 | 0.2299 |
| D(TDP_VALUE_(-1)) | -1.131666 | 0.160374 | -7.056400 | 0.0000 |
| D(TDP_VALUE_(-2)) | -1.044546 | 0.182186 | -5.733404 | 0.0000 |
| D(TDP_VALUE_(-3)) | -0.421047 | 0.159122 | -2.646061 | 0.0108 |
| C | -690978.7 | 1252775. | -0.551559 | 0.5837 |
|  |  |  |  |  |
|  |  |  |  |  |
| R-squared | 0.577663 | Mean dependent var | | 181914.4 |
| Adjusted R-squared | 0.544538 | S.D. dependent var | | 3111347. |
| S.E. of regression | 2099782. | Akaike info criterion | | 32.03761 |
| Sum squared resid | 2.25E+14 | Schwarz criterion | | 32.21845 |
| Log likelihood | -892.0531 | Hannan-Quinn criter. | | 32.10772 |
| F-statistic | 17.43916 | Durbin-Watson stat | | 2.050557 |
| Prob(F-statistic) | 0.000000 |  |  |  |
|  |  |  |  |  |
|  |  |  |  |  |

1. **Stationary test at first difference**

| Null Hypothesis: D(TDP_VALUE_) has a unit root | | | | |
| --- | --- | --- | --- | --- |
| Exogenous: Constant | | | |  |
| Lag Length: 2 (Automatic - based on SIC, maxlag=10) | | | | |
|  |  |  |  |  |
|  |  |  |  |  |
|  |  |  | t-Statistic | Prob.* |
|  |  |  |  |  |
|  |  |  |  |  |
| Augmented Dickey-Fuller test statistic | | | -8.498585 | 0.0000 |
| Test critical values: | 1% level |  | -3.552666 |  |
|  | 5% level |  | -2.914517 |  |
|  | 10% level |  | -2.595033 |  |
|  |  |  |  |  |
|  |  |  |  |  |
| *MacKinnon (1996) one-sided p-values. | | | | |
|  |  |  |  |  |
|  |  |  |  |  |
| Augmented Dickey-Fuller Test Equation | | | | |
| Dependent Variable: D(TDP_VALUE_,2) | | | | |
| Method: Least Squares | | | |  |
| Date: 05/30/25 Time: 21:19 | | | | |
| Sample (adjusted): 2020M02 2024M09 | | | | |
| Included observations: 56 after adjustments | | | | |
|  |  |  |  |  |
|  |  |  |  |  |
| Variable | Coefficient | Std. Error | t-Statistic | Prob. |
|  |  |  |  |  |
|  |  |  |  |  |
| D(TDP_VALUE_(-1)) | -3.323508 | 0.391066 | -8.498585 | 0.0000 |
| D(TDP_VALUE_(-1),2) | 1.302004 | 0.285903 | 4.554012 | 0.0000 |
| D(TDP_VALUE_(-2),2) | 0.362354 | 0.152309 | 2.379081 | 0.0211 |
| C | 787545.0 | 299929.4 | 2.625768 | 0.0113 |
|  |  |  |  |  |
|  |  |  |  |  |
| R-squared | 0.855028 | Mean dependent var | | 7524.904 |
| Adjusted R-squared | 0.846665 | S.D. dependent var | | 5386848. |
| S.E. of regression | 2109386. | Akaike info criterion | | 32.03044 |
| Sum squared resid | 2.31E+14 | Schwarz criterion | | 32.17511 |
| Log likelihood | -892.8524 | Hannan-Quinn criter. | | 32.08653 |
| F-statistic | 102.2302 | Durbin-Watson stat | | 2.013894 |
| Prob(F-statistic) | 0.000000 |  |  |  |
|  |  |  |  |  |
|  |  |  |  |  |

1. **CPI**
2. **Stationary test at level**

| Null Hypothesis: CPI has a unit root | | | | |
| --- | --- | --- | --- | --- |
| Exogenous: Constant | | | |  |
| Lag Length: 2 (Automatic - based on SIC, maxlag=10) | | | | |
|  |  |  |  |  |
|  |  |  |  |  |
|  |  |  | t-Statistic | Prob.* |
|  |  |  |  |  |
|  |  |  |  |  |
| Augmented Dickey-Fuller test statistic | | | 0.686090 | 0.9908 |
| Test critical values: | 1% level |  | -3.550396 |  |
|  | 5% level |  | -2.913549 |  |
|  | 10% level |  | -2.594521 |  |
|  |  |  |  |  |
|  |  |  |  |  |
| *MacKinnon (1996) one-sided p-values. | | | | |
|  |  |  |  |  |
|  |  |  |  |  |
| Augmented Dickey-Fuller Test Equation | | | | |
| Dependent Variable: D(CPI) | | | | |
| Method: Least Squares | | | |  |
| Date: 05/30/25 Time: 21:26 | | | | |
| Sample (adjusted): 2020M01 2024M09 | | | | |
| Included observations: 57 after adjustments | | | | |
|  |  |  |  |  |
|  |  |  |  |  |
| Variable | Coefficient | Std. Error | t-Statistic | Prob. |
|  |  |  |  |  |
|  |  |  |  |  |
| CPI(-1) | 0.008910 | 0.012986 | 0.686090 | 0.4956 |
| D(CPI(-1)) | 0.332726 | 0.129840 | 2.562591 | 0.0133 |
| D(CPI(-2)) | -0.316115 | 0.130366 | -2.424830 | 0.0188 |
| C | -0.749740 | 2.206372 | -0.339807 | 0.7353 |
|  |  |  |  |  |
|  |  |  |  |  |
| R-squared | 0.163090 | Mean dependent var | | 0.778947 |
| Adjusted R-squared | 0.115718 | S.D. dependent var | | 1.373569 |
| S.E. of regression | 1.291653 | Akaike info criterion | | 3.417314 |
| Sum squared resid | 88.42348 | Schwarz criterion | | 3.560686 |
| Log likelihood | -93.39346 | Hannan-Quinn criter. | | 3.473033 |
| F-statistic | 3.442738 | Durbin-Watson stat | | 1.880304 |
| Prob(F-statistic) | 0.023087 |  |  |  |
|  |  |  |  |  |
|  |  |  |  |  |

1. **Stationary test at first difference**

| Null Hypothesis: D(CPI) has a unit root | | | | |
| --- | --- | --- | --- | --- |
| Exogenous: Constant | | | |  |
| Lag Length: 1 (Automatic - based on SIC, maxlag=10) | | | | |
|  |  |  |  |  |
|  |  |  |  |  |
|  |  |  | t-Statistic | Prob.* |
|  |  |  |  |  |
|  |  |  |  |  |
| Augmented Dickey-Fuller test statistic | | | -6.210673 | 0.0000 |
| Test critical values: | 1% level |  | -3.550396 |  |
|  | 5% level |  | -2.913549 |  |
|  | 10% level |  | -2.594521 |  |
|  |  |  |  |  |
|  |  |  |  |  |
| *MacKinnon (1996) one-sided p-values. | | | | |
|  |  |  |  |  |
|  |  |  |  |  |
| Augmented Dickey-Fuller Test Equation | | | | |
| Dependent Variable: D(CPI,2) | | | | |
| Method: Least Squares | | | |  |
| Date: 05/30/25 Time: 21:26 | | | | |
| Sample (adjusted): 2020M01 2024M09 | | | | |
| Included observations: 57 after adjustments | | | | |
|  |  |  |  |  |
|  |  |  |  |  |
| Variable | Coefficient | Std. Error | t-Statistic | Prob. |
|  |  |  |  |  |
|  |  |  |  |  |
| D(CPI(-1)) | -0.962460 | 0.154969 | -6.210673 | 0.0000 |
| D(CPI(-1),2) | 0.303910 | 0.128512 | 2.364838 | 0.0217 |
| C | 0.756979 | 0.211662 | 3.576361 | 0.0007 |
|  |  |  |  |  |
|  |  |  |  |  |
| R-squared | 0.434846 | Mean dependent var | | -0.019298 |
| Adjusted R-squared | 0.413915 | S.D. dependent var | | 1.678907 |
| S.E. of regression | 1.285307 | Akaike info criterion | | 3.391069 |
| Sum squared resid | 89.20882 | Schwarz criterion | | 3.498598 |
| Log likelihood | -93.64546 | Hannan-Quinn criter. | | 3.432858 |
| F-statistic | 20.77462 | Durbin-Watson stat | | 1.866205 |
| Prob(F-statistic) | 0.000000 |  |  |  |
|  |  |  |  |  |
|  |  |  |  |  |

1. **FSI**
2. **Stationarity test at level**

| Null Hypothesis: FSI has a unit root | | | | |
| --- | --- | --- | --- | --- |
| Exogenous: Constant | | | |  |
| Lag Length: 0 (Automatic - based on SIC, maxlag=10) | | | | |
|  |  |  |  |  |
|  |  |  |  |  |
|  |  |  | t-Statistic | Prob.* |
|  |  |  |  |  |
|  |  |  |  |  |
| Augmented Dickey-Fuller test statistic | | | -5.376064 | 0.0000 |
| Test critical values: | 1% level |  | -3.546099 |  |
|  | 5% level |  | -2.911730 |  |
|  | 10% level |  | -2.593551 |  |
|  |  |  |  |  |
|  |  |  |  |  |
| *MacKinnon (1996) one-sided p-values. | | | | |
|  |  |  |  |  |
|  |  |  |  |  |
| Augmented Dickey-Fuller Test Equation | | | | |
| Dependent Variable: D(FSI) | | | | |
| Method: Least Squares | | | |  |
| Date: 05/30/25 Time: 21:24 | | | | |
| Sample (adjusted): 2019M11 2024M09 | | | | |
| Included observations: 59 after adjustments | | | | |
|  |  |  |  |  |
|  |  |  |  |  |
| Variable | Coefficient | Std. Error | t-Statistic | Prob. |
|  |  |  |  |  |
|  |  |  |  |  |
| FSI(-1) | -0.673485 | 0.125275 | -5.376064 | 0.0000 |
| C | -0.142141 | 0.220618 | -0.644284 | 0.5220 |
|  |  |  |  |  |
|  |  |  |  |  |
| R-squared | 0.336454 | Mean dependent var | | -0.002388 |
| Adjusted R-squared | 0.324812 | S.D. dependent var | | 2.047952 |
| S.E. of regression | 1.682798 | Akaike info criterion | | 3.912103 |
| Sum squared resid | 161.4131 | Schwarz criterion | | 3.982528 |
| Log likelihood | -113.4070 | Hannan-Quinn criter. | | 3.939594 |
| F-statistic | 28.90206 | Durbin-Watson stat | | 1.922154 |
| Prob(F-statistic) | 0.000001 |  |  |  |
|  |  |  |  |  |
|  |  |  |  |  |
