# Supplementary material for: Exploring predictive models to improve the accuracy of Housing Price Index forecasts in India’s real estate sector
Source: PLoS One. 2026 Jan 23;21(1):e0341026. doi: 10.1371/journal.pone.0341026 (PMC12829794; doi:10.1371/journal.pone.0341026)
Supplement: S3 File — (DOCX) [file pone.0341026.s003.docx]

**ARIMAX Model**

| Dependent Variable: DHPIM | | | | |
| --- | --- | --- | --- | --- |
| Method: ARMA Maximum Likelihood (OPG - BHHH) | | | | |
| Date: 11/11/25 Time: 23:43 | | | | |
| Sample: 2020M02 2024M09 | | | |  |
| Included observations: 56 | | | | |
| Convergence achieved after 21 iterations | | | | |
| Coefficient covariance computed using outer product of gradients | | | | |
|  |  |  |  |  |
|  |  |  |  |  |
| Variable | Coefficient | Std. Error | t-Statistic | Prob. |
|  |  |  |  |  |
|  |  |  |  |  |
| C | 0.855118 | 0.137592 | 6.214872 | 0.0000 |
| DTDP(-3) | -5.02E-07 | 1.29E-07 | -3.879677 | 0.0003 |
| FSI(-1) | -0.213883 | 0.125677 | -1.701843 | 0.0950 |
| AR(1) | 0.634727 | 0.387961 | 1.636058 | 0.1081 |
| MA(1) | -0.828829 | 0.237146 | -3.495016 | 0.0010 |
| SIGMASQ | 3.488018 | 0.580670 | 6.006887 | 0.0000 |
|  |  |  |  |  |
|  |  |  |  |  |
| R-squared | 0.372943 | Mean dependent var | | 0.746905 |
| Adjusted R-squared | 0.310238 | S.D. dependent var | | 2.379845 |
| S.E. of regression | 1.976507 | Akaike info criterion | | 4.304775 |
| Sum squared resid | 195.3290 | Schwarz criterion | | 4.521777 |
| Log likelihood | -114.5337 | Hannan-Quinn criter. | | 4.388907 |
| F-statistic | 5.947522 | Durbin-Watson stat | | 1.775313 |
| Prob(F-statistic) | 0.000217 |  |  |  |
|  |  |  |  |  |
|  |  |  |  |  |
| Inverted AR Roots | .63 | | |  |
| Inverted MA Roots | .83 | | |  |
|  |  |  |  |  |
|  |  |  |  |  |

**Interpretation:** The ARIMAX model demonstrates a statistically significant lagged effect of digital payment (DTDP(-3)) and a notable short-term impact of the financial stress index (FSI(-1)) on the house price index (DHPIM). As CPI does not show any link with HPI in correlation analysis (Table 1), so it is not included in ARIMAX model. Digital payment data (DTDP) shows moderate positive correlation with DDHPI, suggesting digital financial activity influences household price behavior, making it a key exogenous variable. Including MA(1) enhances the model's fit and residual behaviour. The overall model is significant as the F-statistic falls within the acceptable range (p < 0.001). Inverted AR Roots and Inverted MA Roots are withing the circle (<1) which shows stability of the model. Therefore, this model is appropriate for short-term forecasting and offers valuable insights for policy development.
